# Furin cleavage is required for swine acute diarrhea syndrome coronavirus spike protein-mediated cell – cell fusion

**DOI:** 10.1080/22221751.2022.2114850

**Published:** 2022-09-21

**Authors:** Jinman Kim, Jaewon Yoon, Jung-Eun Park

**Affiliations:** a Laboratory of Veterinary Public Health, College of Veterinary Medicine, Chungnam National University, Daejeon, Republic of Korea; b Research Institute of Veterinary Medicine, Chungnam National University, Daejeon, Republic of Korea

**Keywords:** Swine acute diarrhea syndrome coronavirus, furin, spike, cleavage, cell – cell fusion

## Abstract

Swine acute diarrhea syndrome coronavirus (SADS-CoV) was reported in China in 2017 and is a causative agent of porcine enteric disease. Recent studies indicate that cells from various hosts are susceptible to SADS-CoV, suggesting the zoonotic potential of this virus. However, little is known about the mechanisms through which this virus enters cells. In this study, we investigated the role of furin in SADS-CoV spike (S)-mediated cell – cell fusion and entry. We found that the SADS-CoV S protein induced the fusion of various cells. Cell – cell fusion was inhibited by the proprotein convertase inhibitor dec-RVKR-cmk, and between cells transfected with mutant S proteins resistant to furin cleavage. These findings revealed that furin-induced cleavage of the SADS-CoV S protein is required for cell – cell fusion. Using mutagenesis analysis, we demonstrated that furin cleaves the SADS-CoV S protein near the S1/S2 cleavage site, _446_RYVR_449_ and _543_AVRR_546_. We used pseudotyped viruses to determine whether furin-induced S cleavage is also required for viral entry. Pseudotyped viruses expressing S proteins with a mutated furin cleavage site could be transduced into target cells, indicating that furin-induced cleavage is not required for pseudotyped virus entry. Our data indicate that S cleavage is critical for SADS-CoV S-mediated cell – cell fusion and suggest that furin might be a host target for SADS-CoV antivirals.

## Introduction

Swine acute diarrhea syndrome coronavirus (SADS-CoV) is a novel member of the genus Alphacoronavirus, which was first identified in southern China in 2017 [1–3]. Infection with SADS-CoV induces acute diarrhea and vomiting, similar to porcine epidemic diarrhea, resulting in a high mortality rate among young piglets [1–4]. Bats in a nearby epidemic area were found to harbour coronaviruses (HKU2) carrying 96–98% sequence homology to SADS-CoV, suggesting that bat-derived CoV spilled over to infect pigs [3,5]. Phylogenetic analysis revealed that the S genes of HKU2 and SADS-CoV formed a distinct lineage with all known alphacoronaviruses [3]. SADS-CoV-like CoVs have been identified in 9.8% of tested bats, mainly *Rhinolophus* spp., which are also CoV reservoirs associated with severe acute respiratory syndrome (SARS)-CoV [1,3]. Recent reports have indicated that SADS-CoV can infect cells from several species, including humans [6–8]. These findings highlight the importance of CoV spillover from bats to livestock, and indicate that SADS-CoV carries the potential for interspecies transmission. There are currently no vaccines or antiviral drugs for SADS-CoV [9].

The SADS-CoV genome is ∼ 27 kb long, and contains nine open reading frames (ORFs) along with ORF1a, ORF1b, spike (S), envelope, membrane, nucleocapsid protein-encoding, and three accessory genes (NS3a, NS7a, and NS7b) [3]. S protein mediates viral entry into cells by recognizing cell receptors and catalyzing fusion between viral envelope and the cell membrane [10]. The S protein is divided into three parts: a large ectodomain, a single-pass transmembrane anchor, and a small cytoplasmic tail. The ectodomain of the S protein consists of two domains: the N-terminal receptor-binding domain S1, which is responsible for receptor binding, and the C-terminal membrane fusion domain S2, which is responsible for membrane fusion [11]. The S1 domain contains receptor-binding domains that recognize sugars and proteins on target cells. The S2 domain contains a hydrophobic fusion peptide and two heptad repeats, which are typical features of class I viral fusion proteins [12]. Some CoV S proteins are cleaved by furin/proprotein convertases at a cleavage site between the S1 and S2 domains (the S1/S2 cleavage site) during biosynthesis [13,14]. The S1/S2 cleavage site of CoV S is required for efficient infection and promotes the formation of syncytia [13,14]. Host receptor engagement induces conformational changes in S, exposing another cleavage site adjacent to the hydrophobic fusion peptide (the S2` cleavage site), which is then cleaved by type II transmembrane serine proteases (TTSPs), cathepsins, or furin/proprotein convertases to catalyze membrane fusion [15–17].

Previously, trypsin was shown to enhance virus infection and cell – cell fusion [8], suggesting that proteases, such as trypsin, may trigger SADS-CoV S-mediated membrane fusion. However, the molecular mechanisms underlying the proteolytic priming sites for SADS-CoV S-mediated cell – cell fusion and entry, and protease activity are not entirely clear. In the present study, we have investigated the role of furin in SADS-CoV S-mediated cell-cell fusion. Using specific inhibitors and mutagenesis analysis, we found that furin cleaves the SADS-CoV S protein near the S1/S2 cleavage site and that furin-mediated S cleavage is necessary for cell – cell fusion. These results demonstrate a role for furin in SADS-CoV entry and membrane fusion and indicate that furin is a host target for SADS-CoV control.

## Materials and methods

### Cells

HEK293T (ATCC CRL-3216^TM^), Huh7 (KTCC KCLM60104), Vero81 (ATCC CCL-81^TM^), DBT, IPEC-J2, and Tb1-Lu (ATCC CCL-83^TM^) cells were maintained in Dulbecco’s modified Eagle’s medium supplemented with 10% (v/v) fetal bovine serum, 100 U/mL penicillin, and 100 µg/mL streptomycin. DBT and IPEC-J2 cells were obtained from Hyun-Jin Shin of Chungnam National University (Daejoen, South Korea). All cells were maintained at 37 °C and 5% CO_2_ under water-saturated humidity conditions. Cell culture materials and reagents were obtained from SPL life sciences (Seoul, South Korea) and HyClone (Logan, UT, USA), unless otherwise noted.

### Plasmids

DNA encoding codon-optimized SADS-CoV S (GenBank accession no. MG557844) containing a C-terminal C9 tag was purchased from GenScript (Piscataway, NJ, USA) and cloned into a pCAGGS vector using EcoRI/XhoI restriction enzyme sites. Mutant S proteins were generated by PCR using mutagenic primers (Table 1) and assembled using In-Fusion HD (Clontech, Mountain View, CA, USA). S mutations were confirmed by Sanger sequencing. A plasmid encoding human furin containing a C-terminal myc-DDK tag was purchased from OriGene (Rockville, MD, USA). Plasmids encoding MERS-CoV S containing a C-terminal C9 tag, an enhanced GFP (pEGFP-C1), and pNL4.3-Luc R – E – were obtained from Thomas Gallagher of Loyola University (Maywood, IL, USA).

**Table 1.** Primers used for generating mutant furin cleavage site SADS-CoV S.

Primer name	Primer sequences
AYVA forward	5′ AGCCAGCTGGCCTATGTGGCCATCCTGGGC 3′
AYVA reverse	5′ GCCCAGGATGGCCACATAGGCCAGCTGGCT 3`
KSAA forward	5′ AAGAGCGCCGCCTTCGTGGAT 3′
KSAA reverse	5′ ATCCACGAAGGCGGCGCTCTT 3′
AVAA forward	5′ GTGGCCGTGGCCGCCATGACCTTT 3′
AVAA reverse	5′ AAAGGTCATGGCGGCCACGGCCAC 3′
AYVR forward	5′ AGCCAGCTGGCCTATGTGCGC 3′
AYVR reverse	5′ GCGCACATAGGCCAGCTGGCT 3′
AVAR forward	5′ GTGGCCGTGGCCAGAATGACC 3′
AVAR reverse	5′ GGTCATTCTGGCCACGGCCAC 3′
SADS S1 forward	5′ CATCATTTTGGCAAAGAATTCGCCACC 3′
SADS S1130 reverse	5′ AAAAAGATCTGCTAGCTCGAGTTATGC 3′

### Cell – cell fusion

HEK293T cells were transfected with SADS-CoV S and pEGFP-C1 using polyethylenimine (PEI, Polysciences, Warrington, PA, UK) at a PEI:DNA ratio of 3:1 in Opti-MEM (Life Technologies, Carlsbad, CA, USA) for 15 min at 25 °C. Other cells were transfected using jetPRIME (Polyplus, Strasbourg, France), according to the manufacturer’s instructions. Where indicated, plasmids encoding human furin were co-transfected. Transfected cells were incubated with 5 µg/mL trypsin for 24–48 h post-transfection (hpt). Transfected cells were incubated with protease inhibitors, including 100 µM camostat, 50 µM dec-RVKR-cmk, or 10 µM E64d for 6–48 hpt. All reagents were obtained from Sigma – Aldrich (St Louis, MO, USA), and dimethyl sulfoxide (DMSO) was used as the vehicle control. GFP expression and cell – cell fusion were monitored using a CLENA™ S Digital Imaging System (CronyTek, Anyang, South Korea). Cell – cell fusion was scored as 0, 1, 2, or 3, representing 0%, less than 33%, 33% to 66%, and more than 66% fuzed cells, respectively. Cell nuclei were stained with DAPI and nuclei in the fuzed cells were counted manually.

### HIV pseudotyped virus preparation and transduction

HEK293T cells were transfected with plasmids encoding SADS-CoV S, MERS-CoV S, or an empty vector, along with pNL4.3-Luc R – E-. Cell-free supernatants were collected 48 hpt and passed through a 0.45 µm filter.

The cells were incubated with pseudotyped viruses at normalized inputs based on HIV p24 protein levels for 48 h at 37 °C. Transduced cells were lysed in cell culture lysis buffer (Promega, Madison, WI, USA), and luciferase levels were measured following the addition of Fluc substrate (Promega) using a GloMax® Navigator Microplate Luminometer (Promega).

### Western blot analysis

Transfected cells were lysed on ice in Triton X-100 lysis buffer (1% Triton X-100, 50 mM Tris-Cl [pH 8.0], 150 mM NaCl, 1 mM EDTA) and cleared by centrifugation at 1,000 × g for 10 min at 4°C. Pseudotyped viruses were pelleted by centrifugation at 10,000 × g for 10 h at 4°C. The cell lysates and pelleted pseudotyped viruses were separated in 8% (wt/vol) SDS – PAGE gels, transferred to polyvinylidene difluoride membranes, and probed with monoclonal mouse anti-C9 (EMD Millipore, Burlington, MA, USA) antibody. The membranes were probed with horseradish peroxidase-conjugated goat anti-mouse IgG (Bioss, Woburn, MA, USA) and then incubated with enhanced chemiluminescence substrate (Thermo Fisher Scientific, Waltham, MA, USA). Signals were detected using a Fusion Solo X imaging system (Vilber, Paris, France).

### Statistical analysis

All experiments were independently repeated at least twice. Data are shown as the mean ± standard deviation (SD). Statistical significance was calculated using the Holm – Sidak multiple Student’s t test. A *P* value of <0.05 was considered to be statistically significant.

## Results

### SADS-CoV S protein is cleaved during biosynthesis and induces the fusion of multiple cell types

**Figure 1.**
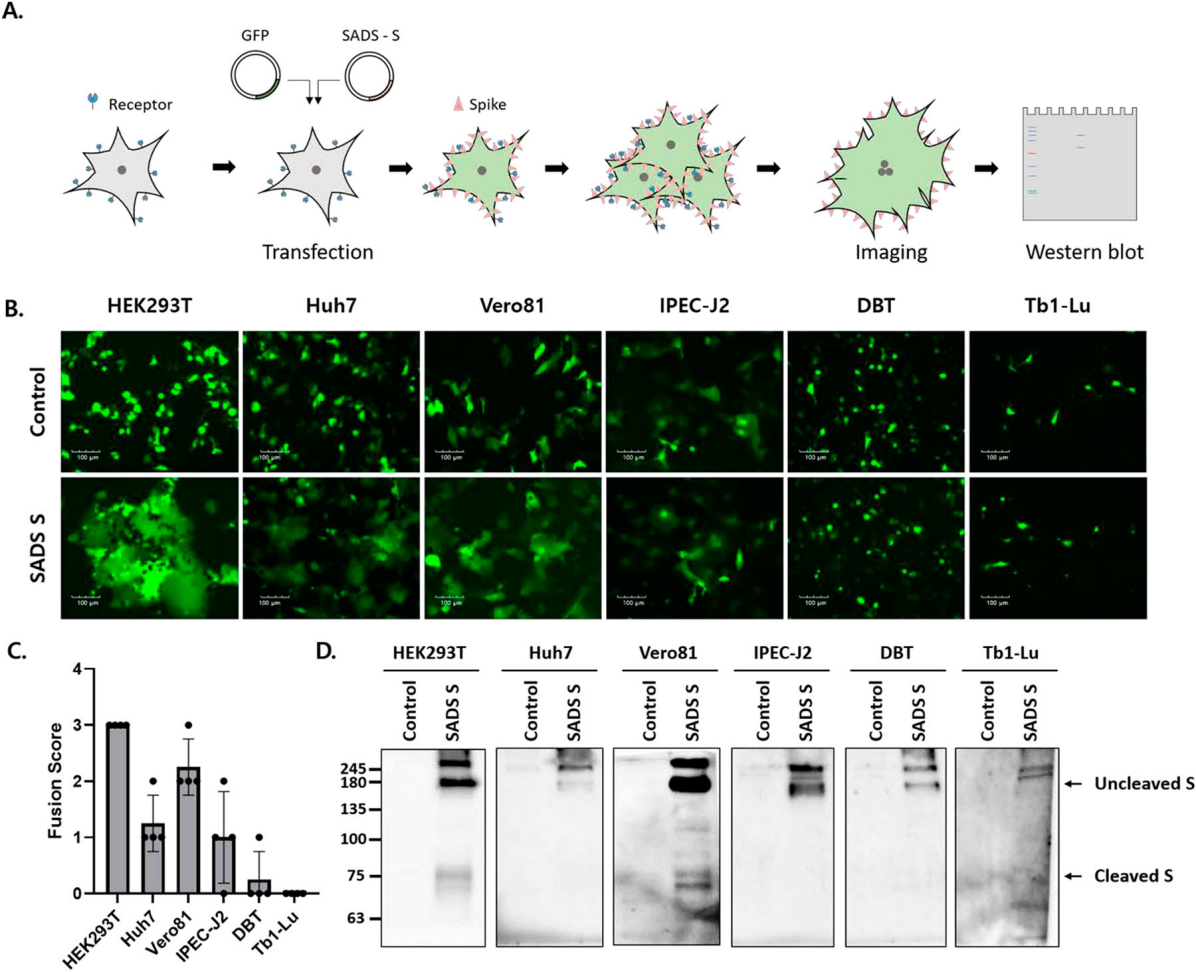
The swine acute diarrhea syndrome coronavirus (SADS-CoV) spike (S) protein induces cell – cell fusion in various cell types. (A) Schematic representation of the cell-cell fusion assay (see details in *Materials and Methods*). (B, C) Cells were transfected with SADS-CoV S-C9 or empty vector (control) and green fluorescent protein (GFP). After 48 h, cell – cell fusion was observed by fluorescence microscopy (B) and scored as 0, 1, 2, or 3, representing 0%, less than 33%, 33% to 66%, and more than 66% fuzed cells (C). Scale bars, 100 µm. (D) S expression was examined by western blot analysis using an anti-C9 antibody.

Genes encoding SADS-CoV S with a C-terminal C9 tag were constructed to evaluate the fusogenic capacity of the SADS-CoV S protein. SADS-CoV S and GFP were co-expressed in six cell lines derived from different animal species (Figure 1A), including human embryonic kidney (HEK293T), human hepatocellular carcinoma (Huh7), African green monkey kidney (Vero81), swine intestinal epithelial (IPEC-J2), mouse delayed brain tumour (DBT), and *Tadarida brasiliensis* lung epithelial (Tb1-Lu) cells. As shown in Figure 1B, GFP expression was diffuse, with a larger area of fuzed cells emitting GFP signals compared to control cells. Prominent cell – cell fusion was observed in HEK293T cells (fusion score = 3 ± 0), moderate cell – cell fusion was observed in Vero81 cells (fusion score = 2.3 ± 0.5), and little cell – cell fusion was observed in Huh7 (fusion score = 1.3 ± 0.5) and IPEC-J2 (fusion score = 1 ± 0.8) cells (Figures 1B and C). No cell – cell fusion was observed in DBT (fusion score = 0.3 ± 0.5) or Tb1-Lu (fusion score = 0) cells (Figures 1B and C).

The expression of SADS-CoV S was assessed by western blotting 48 hpt (Figure 1D). In all transfected cells, SADS-CoV S-specific bands were shifted upward, indicating greater mass than the expected 126 kDa due to the glycosylation of S [18]. S oligomers were also observed in bands representing a mass of ∼245 kDa or greater. An additional band of ∼75 kDa was observed in HEK293T and Vero81 cells exhibiting significant cell – cell fusion; these were most likely fragments resulting from the proteolytic processing of S by host proteases. These data demonstrated that SADS-CoV S proteins induce cell – cell fusion in various cell types and that S cleavage is likely required for SADS-CoV S-mediated cell – cell fusion.

### Furin is required for SADS-CoV S-mediated cell – cell fusion and S cleavage

**Figure 2.**
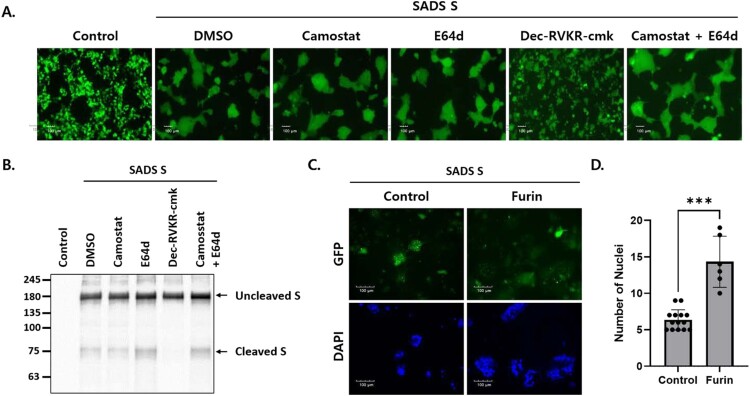
Furin activates SADS-CoV S-mediated cell – cell fusion and S cleavage. (A, B) HEK293T cells were transfected with SADS-CoV S and GFP. After 6 h, transfected cells were treated with the indicated protease inhibitors. Cell – cell fusion was observed by fluorescence microscopy (A). Scale bars, 100 µm. S expression was examined by western blotting (B). (C) Cells were transfected with SADS-CoV S and GFP in the presence or absence of human furin. After 48 h, cell nuclei were stained with DAPI and cell – cell fusion was assessed by fluorescence microscopy. Scale bars, 100 µm. (D) Nuclei in the fuzed cells were counted. Statistical significance was assessed by Student’s t test. ***, *P* < 0.001.

CoV S-mediated membrane fusion is triggered by the proteolytic cleavage of S by host proteases, such as TTSPs [19,20], furin/proprotein convertases [17,21], and endosomal cathepsins [16]. Therefore, we sought to identify the host proteases that mediate SADS-CoV S-mediated cell – cell fusion and S cleavage. Transfected HEK293T cells were incubated with various protease inhibitors. Cell – cell fusion was significantly reduced in cells treated with dec-RVKR-cmk (a subtilisin/Kex2p-like proprotein convertase inhibitor) but was unaffected in cells treated with camostat (a serine protease inhibitor) or E64d (a cysteine protease inhibitor) (Figure 2A). Likewise, no cleaved S proteins were observed in cells treated with dec-RVKR-cmk, but were identified in cells treated with camostat or E64d (Figure 2B).

Dec-RVKR-cmk blocks the activity of proprotein convertases (PCs), including PC1, PC2, PC4, PACE4, PC5, PC7, and furin. These PCs recognize the (R/K)-(2X)n-(R/K)↓ motif and cleave precursor proteins at specific single or paired basic amino acid residues [22]. PCs, particularly furin, have been implicated in viral infections because they cleave host and viral proteins. Specifically, furin has the potential to cleave viral envelope glycoproteins precisely, triggering membrane fusion between the viral envelope and host cell membranes [23]. To determine whether exogenous expression of furin induces cell – cell fusion, Huh7 cells were transfected with SADS-CoV S protein and human furin. As expected, exogenous furin expression increased cell – cell fusion of Huh7 cells (Figures 2C and D). These data indicated that furin proprotein convertases are critical for SADS-CoV S cleavage and cell – cell fusion.

### S cleavage at two furin cleavage sites is required for cell – cell fusion

**Figure 3.**
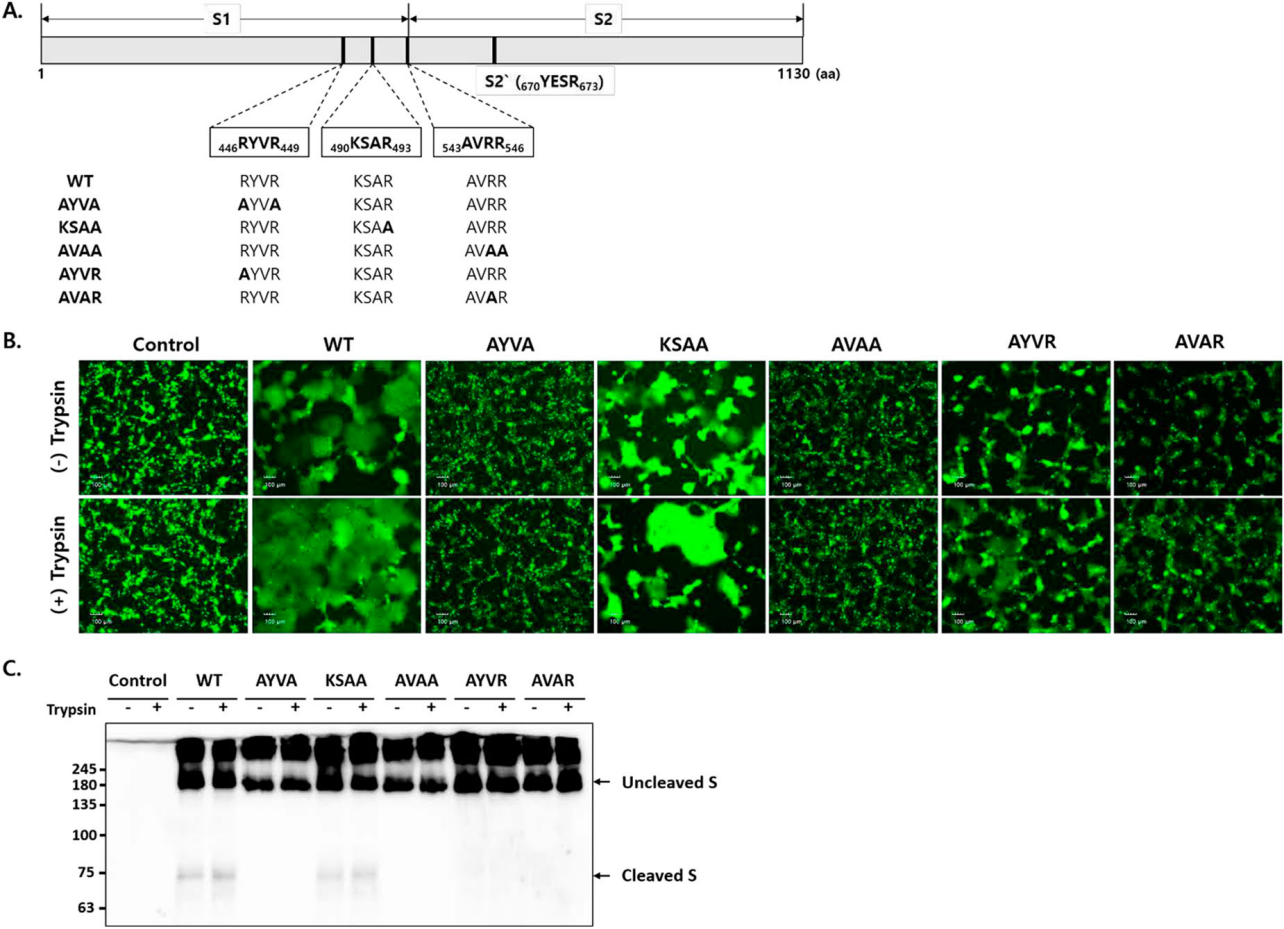
S cleavage at two furin cleavage sites is required for cell – cell fusion. (A) Schematic showing the SADS-CoV S protein containing a receptor binding domain (S1) and a fusion domain (S2). The amino acid (aa) residues of three putative furin cleavage sites and the S2` cleavage sites are indicated, and the mutations introduced into the cleavage sites are shown in bold. (B) HEK293T cells expressing wild type (WT) and mutant S proteins along with GFP. Cell – cell fusion was observed by fluorescence microscopy. Where indicated, cells were treated with 5 µg/mL trypsin. Scale bars, 100 µm. (C) S expression was examined by western blotting.

CoV S possesses two cleavage sites: S1/S2 and S2` [24]. With the expectation that furin cleaves SADS-CoV S proteins at the S1/S2 cleavage site, the furin cleavage motifs were predicted using ProP software [25]. However, no strong furin cleavage motifs were identified in SADS-CoV S. Therefore, we manually searched for furin cleavage motifs and selected three putative furin cleavage sites at the boundary of S1 and S2: _446_RYVR_449_, _490_KSAR_493_, and _543_AVRR_546_ (Figure 3A). To assess whether these cleavage sites in the S protein are cleaved by furin, three furin cleavage site mutants were engineered in which arginine codons were replaced with alanine codons: _446_AYVA_449_, _490_KSAA_493_, and _543_AVAA_546_. To determine whether S1/S2 cleavage is sufficient for membrane fusion, additional mutant S proteins were generated with arginine replacements at the following cleavage sites: _446_AYVR_449_ and _543_AVAR_546_. These mutant S proteins were expected to be expressed in uncleaved forms and cleaved by exogenous trypsin.

We compared the fusogenic capacity of wild-type (WT) and mutant S proteins in HEK293T cells. Fusion was observed between cells transfected with the WT and KSAA mutants, but not between cells transfected with the AYVA and AVAA mutants (Figure 3B). In cells transfected with AYVR and AVAR S proteins, low levels of cell – cell fusion were observed in the absence of trypsin, whereas exogenous trypsin increased the rate of fusion (Figure 3B). S protein expression was examined by western blot analysis (Figure 3C). Cleaved S fragments of ∼75 kDa were observed in cells transfected with WT and KSAA mutant S proteins, but not in cells transfected with AYVA, AVAA, AYVR, and AVAR S proteins. These data indicated that the SADS-CoV S protein is cleaved by furin at two sites, namely, _446_RYVR_449_ and _543_AVRR_546_, and that S cleavage at both furin cleavage sites is required for cell – cell fusion.

### SADS-CoV S cleavage by furin is not required for viral entry

**Figure 4.**
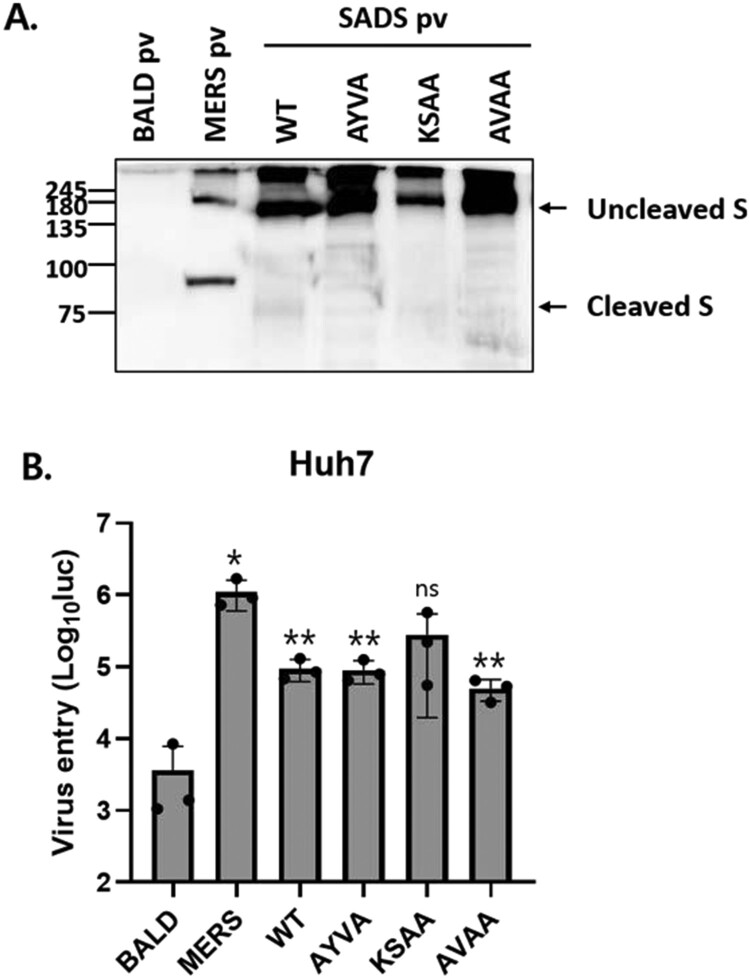
SADS-CoV S cleavage by furin is not required for viral entry. (A) S proteins in pseudotyped viruses (pv) were examined by western blotting. BALD pv indicates pseudotyped viruses lacking S proteins. (B) Huh7 cells were transduced with the indicated pseudotyped viruses. After 48 h, viral entry was quantified by measuring luciferase levels. Statistical significance was assessed by Student’s t test. *P < 0.05; **P < 0.01; ***P < 0.001; ns, not significant.

To examine the role of furin cleavage in viral entry, we produced HIV-based pseudotyped viruses expressing WT or mutant S proteins. First, pseudotyped virus-associated S protein was evaluated by western blot analysis (Figure 4A). In all pseudotyped viruses, the S protein was primarily expressed in an uncleaved form, and few cleaved S proteins were observed. S expression in WT and KSAA pseudotyped viruses was slightly reduced, likely due to the high levels of cell – cell fusion. Pseudotyped viruses were incubated with cells and viral entry was measured using a luciferase assay. Huh7 cells were transduced with both WT and mutant pseudotyped viruses (Figure 4B). The results indicated that furin cleavage of SADS-CoV S is not required for viral entry.

## Discussion

In the present study, we demonstrated a role for furin/proprotein convertases in SADS-CoV S-mediated cell – cell fusion. We showed that the SADS-CoV S protein is expressed in a cleaved form and markedly increases the fusion of human cells. Using specific inhibitors and cleavage site mutant S proteins, furin was found to cleave the S protein at two cleavage sites. Furthermore, cleavage at both sites was found to be required for cell – cell fusion. We generated pseudotyped SADS-CoVs expressing WT and mutant S proteins, and found that furin-mediated S cleavage is not necessary for viral entry into target cells. To our knowledge this is the first report to describe a role for host proteases in SADS-CoV S cleavage and membrane fusion.

The importance of furin-mediated CoV S processing in viral entry and cell – cell fusion has been well characterized. In CoVs, such as SARS-CoV-2 [26], Middle East respiratory syndrome coronavirus (MERS-CoV) [17], feline infectious peritonitis virus (FIPV) [27], infectious bronchitis virus (IBV) [21], and murine hepatitis virus (MHV) [13], furin cleaves the S protein at the S1/S2 cleavage site to generate S1 and S2 subunits. Furin-mediated S1/S2 cleavage of these CoV S proteins is required for efficient cell – cell fusion [13,28,29]. Although S1/S2 cleavage by furin is important in S-mediated cell – cell fusion, studies have shown that furin-mediated S1/S2 cleavage is not required for viral entry into cells [13,28,29], likely because S proteins can be cleaved by other host proteases during cell entry. Consistent with previous reports, our results indicated that furin-mediated S cleavage is not required for viral entry into target cells.

Furin is a ubiquitously expressed and widely distributed protease [30]. A phylogenetic analysis showed that human furin shares 94.86 and 93.83% similarity with porcine and murine furin, respectively, suggesting high interspecies similarity of this protease. Therefore, CoVs with S-containing furin cleavage sites may confer advantages for both spreading and zoonotic adaptation. Mutations in furin cleavage sites are more common in viruses cultured *in vitro* than in those isolated from clinical samples, suggesting that this cleavage site is subjected to selection pressure in humans [31,32]. In addition, it mutations near the furin cleavage site in S proteins affect cellular tropism and pathogenesis [14,27,33]. Cleavage of S by furin mediates MERS-CoV infection in lung epithelial cells by facilitating the conformational changes and entry kinetics that enable viral entry [14]. Deletion of the furin cleavage site in SARS-CoV-2 was found to attenuate viral replication in respiratory cells and subsequent pathogenesis in hamsters [31,34,35]. Previous studies have demonstrated the zoonotic potential of SADS-CoV [6–8], but the mechanism underlying zoonosis is unknown. The results of our study suggest that furin may allow SADS-CoV to infect a wide range of hosts; however, this needs to be validated in conjunction with receptor specificity.

CoV S proteins are proteolytically processed at two cleavage sites, between the S1 and S2 domains (S1/S2 cleavage site) and immediately before the fusion peptide (S2` cleavage site), during S biosynthesis and/or viral entry [36]. As noted, furin cleaves CoV S proteins once at the S1/S2 cleavage site (in SARS-CoV-2, MERS-CoV, FIPV, IBV, and MHV) or twice at the S1/S2 and S2` cleavage sites (in MERS-CoV). Our results indicated that the SADS-CoV S protein is cleaved at two sites. One cleavage site (RYVR) is located 97 amino acids upstream of the S1/S2 cleavage site, and the other (AVRR) at the S1/S2 cleavage site (Figure 3A). Interestingly, mutations of each cleavage site inhibited both S-mediated cell fusion and S cleavage (Figure 3), suggesting that the S protein needs to be cleaved at both sites for successful S cleavage and S-mediated cell fusion. Similar to other CoV S proteins, the SADS-CoV S protein contains conserved internal fusion peptide sequences at 674–681 aa. Therefore, we speculate that an additional cleavage site may be present in the SADS-CoV S protein (YESR, the S2` cleavage site, shown in Figure 3A), suggesting that the SADS-CoV S protein contains at least three cleavage sites. In fact, recent studies have shown that SARS-CoV-2 and MERS-CoV S proteins have cleavage sites in addition to the S1/S2 and S2` sites [26,37]. These sites are reportedly located between the S1/S2 and S2` cleavage sites and are targeted by cathepsin L [26,37]. Further studies are needed to determine how multiple cleavages participate in CoV S-mediated virus entry and membrane fusion.

We observed prominent cell – cell fusion in cultured cells. The importance of cell – cell fusion in SADS-CoV pathogenesis *in vivo* is not known. However, cell – cell fusion may be important for CoV pathogenesis, as it mediates virion-free transmission [38] and promotes histopathological changes during SARS-CoV-2 infection [39,40]. Thus, further studies are needed to investigate the role of cell – cell fusion in SADS pathogenesis.

Taken together, our results demonstrated the presence of two furin cleavage sites in SADS-CoV S, both of which are essential for cell – cell fusion. This is a novel observation that has not, to our knowledge, been previously reported for SADS-CoV or other identified CoVs. Our research provides important data for understanding the mechanism through which SADS-CoV enters cells, and for the development of prophylactic and therapeutic agents for SADS-CoV infection.
